# Identification, Isolation, and Molecular Characterization of *Betacoronavirus* in *Oryx leucoryx*

**DOI:** 10.1128/spectrum.04848-22

**Published:** 2023-07-10

**Authors:** Dan David, Jimmy Asiku, Nick Storm, Katya Lapin, Asaf Berkowitz, Anita Kovtunenko, Nir Edery, Roni King, Asaf Sol

**Affiliations:** a Kimron Veterinary Institute, Beit Dagan, Israel; b The Hebrew University of Jerusalem, Jerusalem, Israel; c Israel Nature and Parks Authority, Jerusalem, Israel; Erasmus MC

**Keywords:** coronaviruses, diarrhea, spike glycoprotein, animal disease, *Oryx leucoryx*

## Abstract

Coronaviruses (CoVs) are enveloped viruses with a large RNA genome (26 to 32 kb) and are classified into four genera: *Alphacoronavirus*, *Betacoronavirus*, *Gammacoronavirus,* and *Deltacoronavirus*. CoV infections cause respiratory, enteric, and neurologic disorders in mammalian and avian species. In 2019, Oryx leucoryx animals suffered from severe hemorrhagic diarrhea and high morbidity rates. Upon initial diagnosis, we found that the infected animals were positive for coronavirus by pancoronavirus reverse transcriptase RT-PCR. Next, we detected the presence of CoV particles in these samples by electron microscopy and immunohistochemistry. CoV was isolated and propagated on the HRT-18G cell line, and its full genome was sequenced. Full-genome characterization and amino acid comparisons of this viral agent demonstrated that this virus is an evolutionarily distinct *Betacoronavirus* belonging to the subgenus *Embecovirus* and the *Betacoronavirus 1* species. Furthermore, we found that it is most similar to the subspecies dromedary camel coronavirus HKU23 by phylogenetic analysis. Here, we present the first report of isolation and characterization of *Betacoronavirus* associated with enteric disease in *Oryx leucoryx.*

**IMPORTANCE** CoVs cause enteric and respiratory infections in humans and animal hosts. The ability of CoVs to cross interspecies barriers is well recognized, as emphasized by the ongoing pandemic of severe acute respiratory syndrome coronavirus 2 (SARS-CoV-2). The identification of novel CoV strains and surveillance of CoVs in both humans and animals are relevant and important to global health. In this study, we isolated and characterized a newly identified *Betacoronavirus* that causes enteric disease in a wild animal, *Oryx leucoryx* (the Arabian oryx). This work is the first report describing CoV infection in *Oryx leucoryx* and provides insights into its origin.

## INTRODUCTION

Coronaviruses (CoVs) are enveloped positive-strand RNA viruses with large genomes ranging between 26.4 and 31.7 kb (1). CoVs belong to the *Coronaviridae* family in the order *Nidovirales* and are divided into four genera, *Alphacoronavirus*, *Betacoronavirus*, *Gammacoronavirus*, and *Deltacoronavirus*, based on phylogeny analysis (2). Current molecular characterizations of *Betacoronavirus* subdivided this genus into five subgenera, namely *Sarbecovirus*, *Embecovirus*, *Merbecovirus*, *Nobecovirus*, and *Hibecovirus* (2). The viruses of the *Embecovirus* subgenus have a hemagglutinin esterase (HE) protein in addition to the other major structural proteins found in CoVs, membrane (M), envelope (E), nucleocapsid (N), and spike (S) (3, 4). CoVs cause respiratory, enteric, and neurologic diseases in humans and animals (5, 6) and are responsible for several emerging diseases in recent years, including severe acute respiratory syndrome coronavirus (SARS-CoV), Middle Eastern respiratory syndrome coronavirus (MERS-CoV), and the ongoing SARS-CoV-2 pandemic (7, 8). Recent emerging CoVs in the *Betacoronavirus* genus include the identification of a novel betacoronavirus in humans and in domestic and wild animals (8–10). The emergence of novel CoVs requires the ability to adapt to a new host’s environment, which is attainable mostly by the high rates of homologous RNA recombination events (11). CoV host expansion, tissue tropism, and spillover from animals to humans are well documented (12, 13).

The Arabian oryx (Oryx leucoryx) belongs to the *Bovidae* family and is listed as a critically endangered species by the International Union for the Conservation of Nature (14). The oryx herd described in this study is a breeding nucleus that roams in a few square kilometers in southern Israel. This herd includes 131 individuals and serves as a successful reintroduction project in the Negev Desert (14). From January to March 2019, 37 individuals in this herd (28%) faced a unique outbreak of digestive system syndrome, first identified by the occurrence of bloody patches on the hind legs and abnormal lethargic behavior. In a few days, hemorrhagic and nonhemorrhagic diarrhea cases appeared that resembled typical clinical signs of enteric disease as seen for other CoVs, such as bovine coronavirus (BCoV) and bovine-like coronavirus, in wild ruminants (9, 15). Most of the sick animals recovered in a few days from the appearance of the disease. Based on these clinical signs, we aimed to uncover this enteric pathogen.

Here, we describe the isolation and molecular characterization of *Betacoronavirus* virus associated with enteric disease in the *Oryx leucoryx* herd.

## RESULTS

### Molecular identification of coronavirus in oryx fecal samples.

**FIG 1:**
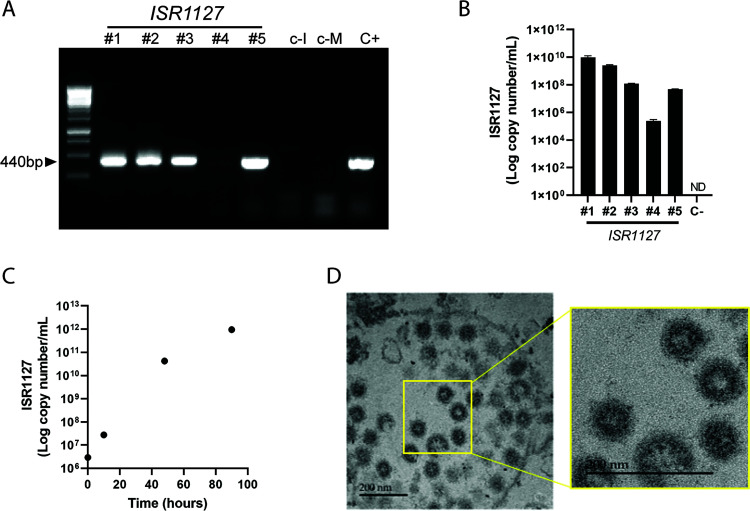
Identification and isolation of coronavirus from *Oryx leucoryx*. (A) Pan-coronavirus nested PCR targeting the RdRp gene by amplifying a final product of 440 bp. RNA was isolated from five oryx fecal samples, ISR1127 samples 1 to 5. c-I, negative control for RNA isolation; c-M, negative control for PCR; C+, positive control, bovine coronavirus RNA. (B) Purified RNA from ISR1127 samples 1 to 5 was used to perform RT-qPCR for BCoV detection. (C) Replication of ISR1127 in HRT-18G cells. Cells were infected with isolate ISR1127 (sample 1) and harvested at different time points, and replication was measured by BCoV RT-qPCR. (D) TEM images showing CoV particles in infected HRT-18G cells at 48 h postinfection. The area of interest outlined in yellow in the left panel is enlarged in the right panel; scale bar, 200 nm.

Based on clinical signs of the sick animals, showing hemorrhagic diarrhea, and due to negative results obtained from routine tests to detect other viral agents (such as bluetongue virus, bovine viral diarrhea virus, and foot-and-mouth disease virus, etc.), we decided to perform a pancoronavirus nested PCR for the detection of CoVs. To this end, we isolated RNA from five fecal and rectal swab samples (ISR1127; described in Materials and Methods) and performed nested PCR for the amplification of the RNA-dependent RNA polymerase (RdRp) gene as previously described (16). Our results showed that in four of five oryx fecal samples tested, we detected the RdRp gene amplicon of 440 bp (Fig. 1A). We further gel purified these products and subjected them to sequencing. BLAST analysis of these sequences showed that all samples were most similar to BCoV. To verify our results, we ran a specific one-step reverse transcriptase quantitative PCR (RT-qPCR) for the detection of the BCoV nucleocapsid gene, and all samples were positive (Fig. 1B). Based on our results, we measured the prevalence of BCoV in 50 samples, from 43 symptomatic animals, collected between 2019 and 2021. We found that 33% of the tested animals were positive for BCoV (see Table S1 in the supplemental material). Most of the positive samples were collected between January and March 2019, supporting the observation of an outbreak event early in 2019 (Table S1). Overall, during this period, 15 animals died, 8 of them during the outbreak in 2019 and 7 between 2020 and 2021 (Table S1). There was no defined age pattern for the sick or dead animals, mature as well as young, and we could not find any correlation between morbidity or mortality and animal gender.

To our knowledge, this is the first time a coronavirus associated with an enteric disease has been described in an *Oryx leucoryx* herd.

### Isolation of coronavirus associated with enteric disease in *Oryx leucoryx*.

To further characterize this coronavirus causing enteric disease in oryx, we isolated this viral agent on the HRT-18G cell line. To this end, we filtered fecal samples and diluted them with phosphate-buffered saline (PBS). We used diluted ISR1127 to infect the HRT-18G monolayer, and cells were observed daily for morphological changes and the development of cytopathic effect (CPE). As for other BCoV strains, we did not observe a clear and significant CPE in infected cells. Therefore, we decided to monitor viral replication by one-step RT-qPCR. As shown in Fig. 1C, log copy numbers of CoVs targeting RNA increased throughout the infection. This result indicates that coronavirus isolated from *Oryx leucoryx* samples replicates efficiently *in vitro* in the HRT-18G cell line (Fig. 1C). These results are consistent with previous studies describing CoV replication in HRT-18G (15, 17, 18). To further verify that ISR1127 is indeed a coronavirus, we used electron microscopy (EM) and analyzed HRT-18G cells infected with ISR1127. We tested both the cell supernatant and the cell pellet for CoV particles. Our EM images confirmed that ISR1127 is a coronavirus, with particle sizes in the range described for CoVs (Fig. 1D) (6). These results are in line with the clinical signs and molecular diagnosis described above (Fig. 1A to C).

### Detection of coronavirus in tissue sections of *Oryx leucoryx*.

**FIG 2:**
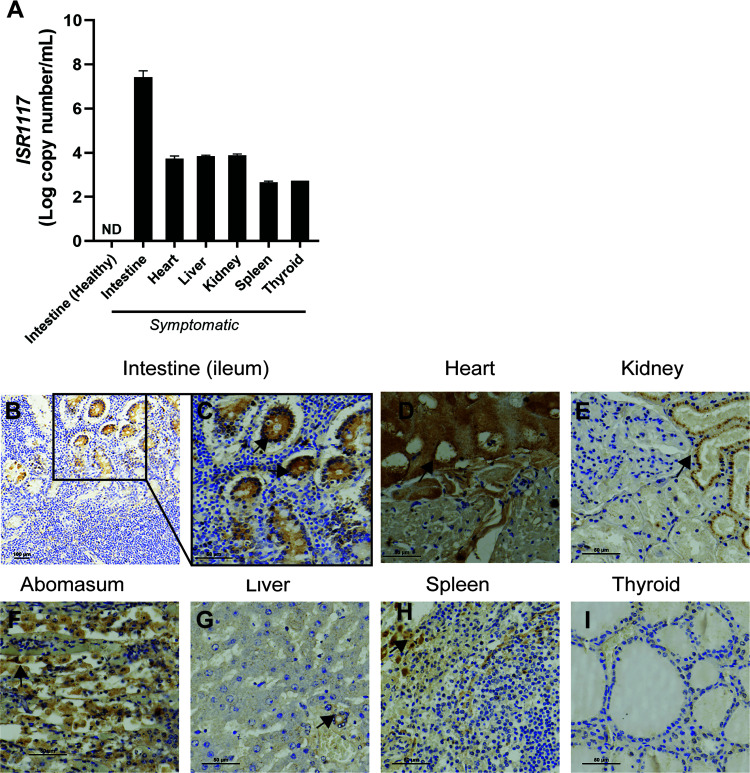
Detection of oryx coronavirus in tissue sections. (A) Identification of coronavirus RNA in FFPE sections from oryx tissues. RNA isolated from oryx tissue section sample ISR1117 was used for BCoV RT-qPCR to determine viral copy number in target tissues. (B to I) IHC analysis for oryx coronavirus in tissue cross sections of sample ISR1117 with an antinucleocapsid antibody. (B) Low magnification of the mucosa of the ileum (small intestine) exhibiting a strong positive cytoplasmic IHC labeling of oryx coronavirus in mucosal epithelial cells. Magnification, ×40; scale bar, 100 μm. (C) Section outlined in panel B; black arrows indicate positive regions. (D) Cross section of the heart exhibiting strong IHC labeling within a bundle of Purkinje fibers (black arrow). (E) Kidney cross section showing IHC labeling of the epithelium of the distal convoluted tubules (black arrow). (F) Abomasum cross section exhibiting a strong positive IHC labeling within epithelial cells. (G) Cross section of the liver exhibiting weak positive IHC labeling within bile duct epithelium (black arrow). (H) Cross section of the spleen exhibiting moderate to strong positive IHC labeling of presumptive histiocytes. (I) Thyroid staining exhibiting negative to very weak IHC labeling of the thyroidal follicular cells. (C to I) Magnification, ×200; scale bar. 50 μm; IHC with hematoxylin counterstain.

Our *in vitro* work suggested that coronavirus was the causative agent of severe diarrhea in oryx animals in an outbreak in early 2019 (Fig. 1A to D). To support this, we performed RNA isolation from formalin-fixed, paraffin-embedded (FFPE) tissues that were collected from an animal that suffered from hemorrhagic diarrhea and eventually died during the 2019 outbreak (termed ISR1117) (Table S1). Isolated RNA was used to detect viral RNA by one-step RT-qPCR. Our results showed that coronavirus RNA could be detected in several tissues but is found mostly in animal intestines (Fig. 2A), which correlated with the clinical signs observed in these animals and is consistent with other CoV infections known to cause enteric disease in animals and humans (19, 20). Furthermore, to assess viral tropism, we performed immunohistochemistry (IHC) on representative tissue sections from ISR1117 (Table S1; Fig. 2B to I). Our IHC images show significant detection of coronavirus in multiple oryx organs (Fig. 2B to I). We observed a strong positive signal of coronavirus antigen in the mucosal epithelial cells of the small intestine (Fig. 2B and C, black arrows) and moderate labeling among inflammatory cells in the lamina propria and a lymphocytic aggregate of the gut-associated lymphoid tissue (GALT), including Peyer’s patches (Fig. 2B and C). In the heart, we observed strong IHC labeling within a bundle of Purkinje fibers, the subendocardial conduction system (Fig. 2D, black arrow), along with relatively weak labeling within the myocardial cells (Fig. 2D). In addition, we detected coronavirus labeling in the kidney, mostly within the epithelium of the distal convoluted tubules of the kidney (Fig. 2E, black arrow) and within the epithelial cells in the mucosa of the abomasum (Fig. 2F). In a cross section of the liver, we observed positive labeling mostly confined to the bile duct epithelium (Fig. 2G, black arrow), and in the spleen, positive IHC labeling was detected in presumptive histiocytes (Fig. 2H, black arrow) with a relatively weak signal within the lymphocytes comprising the lymphoid follicles (Fig. 2H). A cross section of the thyroid exhibited negative to very weak labeling of the thyroidal follicular cells (Fig. 2I). Overall, these results, along with coronavirus RNA detection in FFPE blocks (Fig. 2A), suggest that while the gastrointestinal tract serves as a major target of the oryx coronavirus, infection of additional tissues such as the heart and kidney is possible (Fig. 2), which is consistent with other CoV infections, such as that with SARS-CoV-2 (21, 22).

### Molecular characterization of *Oryx leucoryx* coronavirus.

**FIG 3:**
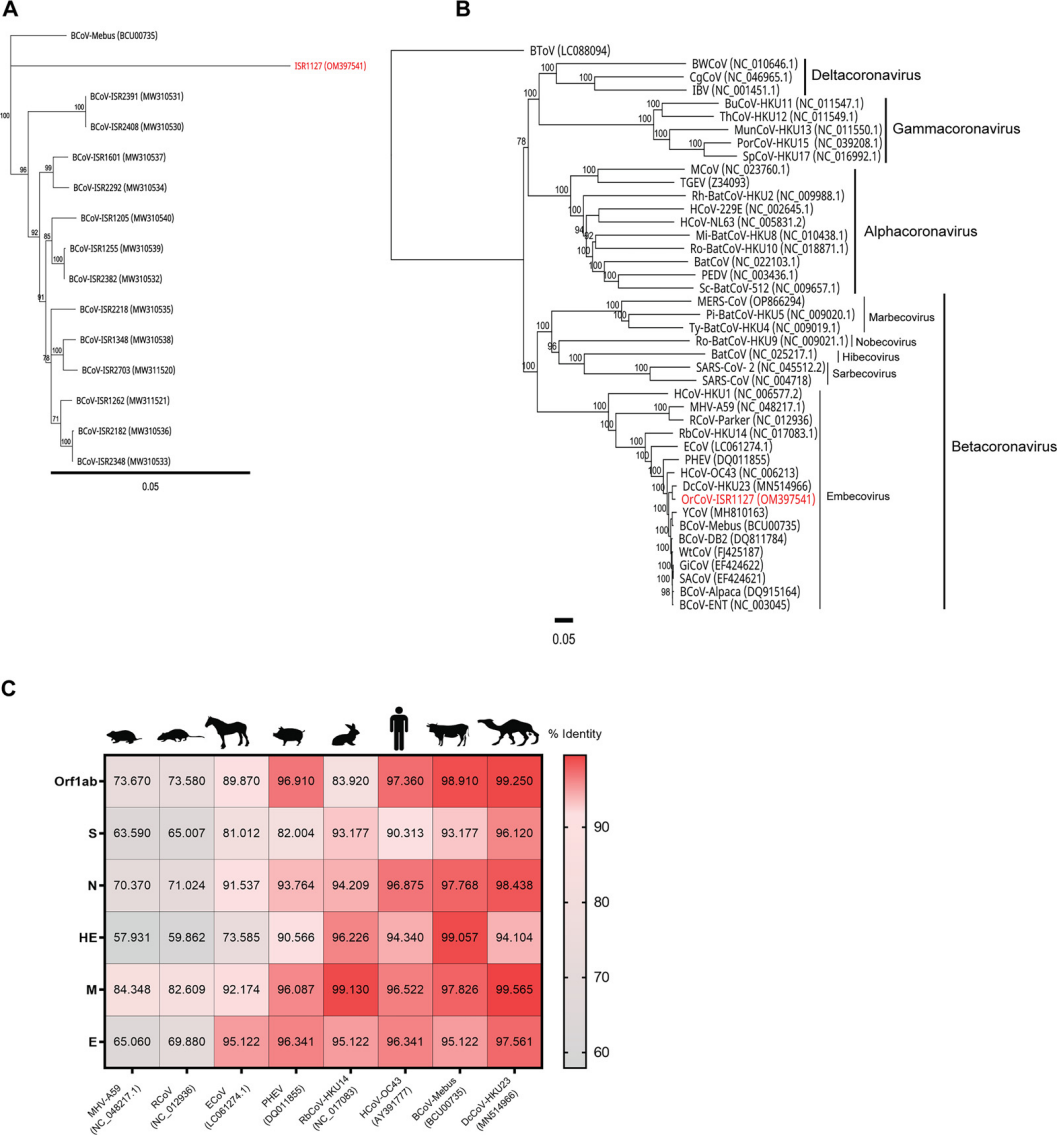
Phylogenetic analysis and amino acid comparison of the newly identified *Betacoronavirus* from *Oryx leucoryx*. (A) Phylogenetic tree of the S1 region of local BCoV strains and *Oryx leucoryx* sample ISR1127 (highlighted in red). A phylogenetic tree was generated based on S1 nucleotide sequences (nucleotides 1 to 2731) using the neighbor-joining method with bootstrap analysis (1,000 replicates, >70%). The scale bar shows the number of substitutions per site. Spike partial sequences were retrieved from GenBank and have been previously published (3). The accession number for each strain is shown in parentheses. (B) Phylogenetic analysis of the full genome of representative coronaviruses. *Oryx leucoryx* sample ISR1127 is highlighted in red. A phylogenetic tree was generated based on full genomes using the neighbor-joining method with bootstrap analysis (1,000 replicates, >70%). Full-genome sequences were retrieved from GenBank, and their accession numbers are shown in parentheses. Phylogenetic trees were generated using Geneious Prime software. (C) Amino acid comparison of *Embecovirus* ORF1ab and structural proteins. *Oryx leucoryx* sample ISR1127 was used as a reference. Numbers and colors in the heat map represent the percent identities between selected strains and *Oryx leucoryx* coronavirus. Pairwise identity was generated using Geneious Prime software, and the heat map was generated in Prism software.

We initially focused on the molecular characterization of the S1 region of the spike protein of sample ISR1127. To this end, we used a previously described set of primers, with slight modifications to ensure amplification of the ISR1127 S1 region (Table S1) (23). We then purified and sequenced the PCR product. Phylogenetic analysis comparing the S1 region of sample ISR1127 with the S1 region of local BCoV strains previously described by us (15) suggested that ISR1127 is genetically distinct from BCoV strains circulating in the area (Fig. 3A). Further BLAST analysis showed that the S1 region of sample ISR1127 is similar to the S1 region of the dromedary camel coronavirus HKU23 (DcCoV-HKU23; GenBank accession no. MN514966). Based on this result, we decided to perform next-generation sequencing and sequence the full genome of *Oryx leucoryx* coronavirus sample ISR1127. To this end, we isolated RNA from HRT-18G cells infected with ISR1127 for 48 h and performed RNA sequencing. Raw reads were mapped to DcCoV-HKU23 (GenBank accession no. MN514966), and the full genome was assembled and deposited in the NCBI database (GenBank accession no. OM397541) (Fig. S2A). Next, we performed a phylogenetic analysis with the full genome of ISR1127 and compared it to selected CoVs, representing different genera (Fig. 3B). Results showed that oryx coronavirus (GenBank accession no. OM397541) is more similar to DcCoV than BCoV and bovine-like CoVs (Fig. 3B), with an overall genome identity of 97.93%. We then performed an amino acid comparison of coronavirus Orf1ab and its major structural proteins (Fig. 3C). Amino acid alignments of ISR1127 major structural proteins spike (S), nucleocapsid (N), membrane (M), and envelope (E) and Orf1ab with other members of the subgenus *Embecovirus* showed that ISR1127 is more similar to DcCoV than to the other embecoviruses, with pairwise amino acid identities of 96.12%, 98.43%, 99.56%, 97.56%, and 99.25%, respectively (Fig. 3C). Interestingly, the HE of ISR1127 is most similar to that of BCoV, with 99.05% identity (Fig. 3C). Next, to determine the genus and species that oryx coronavirus belongs to, we followed ICTV criteria (2). To this end, we performed pairwise amino acid identity analysis of conserved domains of selected CoV replicase proteins with oryx coronavirus sample ISR1127 (Table 1). Our results show that oryx coronavirus ISR1227 is a member of the *Betacoronavirus* genus, subgenus *Embecovirus*, and belongs to the *Betacoronavirus 1* species (Table 1). These data support the phylogenetic analysis showing that oryx coronavirus is most similar to DcCoV-HKU23 (Fig. 2B and C).

**TABLE 1:** Pairwise amino acid identity of replicase conserved domains of OrCoV/ISR1127 with representative coronaviruses^*a*^

Coronavirus	Accession no.	% Identity with OrCoV/ISR1127						
		ADRP	NSP 5-3CLpro	NSP 12-RdRp	NSP 13-Helicase	NSP 14-ExoN	NSP 15-NendoU	NSP 16-2′-O-MT
HCoV-229E	WDE20190	29.46	44.55	55.22	59.71	51.54	48.03	55.37
Bat coronavirus	QBP43288	34.11	42.76	57.48	56.76	51.16	50.66	53.36
DcCoV-HKU23	MN514966	98.4	99.66	99.35	99.71	100	98.01	99.66
BCoV	NP_150073	99.2	100	99.57	99.71	99.81	98.68	100
HCoV-OC43	AGT51647	98.4	98.3	98.16	99.41	98.45	98.68	99.32
MERS-CoV	QEJ82224	44.8	53.54	67.42	68.24	57.69	54.97	62.84
SARS-CoV-2	QNO98001	34.4	41.3	66.10	68.24	58.02	54.3	66.22
Infectious bronchitis virus	WBY69735	36.36	40.13	58.97	58.72	54.81	37.66	53.67
PorCoV	QWX20056	28.68	37.5	51.33	50.58	40.34	35.95	39.71

a HCoV, human coronavirus; DcCoV, dromedary camel coronavirus; BCoV, bovine coronavirus; MERS-CoV, Middle East respiratory syndrome coronavirus; SARS-CoV-2, severe acute respiratory syndrome coronavirus 2; PorCoV, porcine coronavirus; ADRP, ADP-ribose-1′′-phosphatase; 3CLpro, 3C-like protease; RdRp, RNA-dependent RNA polymerase; ExoN, exoribonuclease; NendoU, nidoviral uridylate-specific endoribonuclease; 2′-O-MT, 2′-*O*-methyltransferase.

### Genome organization of oryx coronavirus.

**FIG 4:**
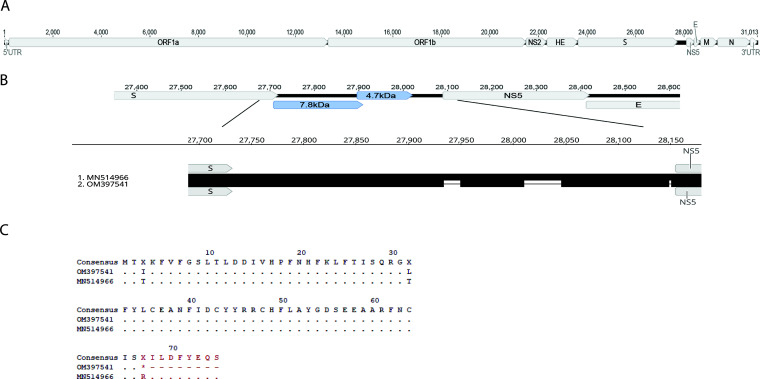
Genomic organization of *Oryx leucoryx* coronavirus. (A) Full-genome organization of OrCoV/ISR1127 (GenBank accession no. OM397541). The scheme shows genome size and major ORF positions. (B) Genomic organization of the region between the S gene and NS5. The scheme shows distinct ORFs in this region of 7.8 kDa and 4.7 kDa. Alignment with DcCoV (accession no. MN514966) in this region shows two deletion domains in OrCoV/ISR1127 (black lines). (C) Deduced amino acid comparison of an OrCoV/ISR1127 (accession no. OM397541) ORF encoding a 7.8-kDa protein with a DcCoV (accession no. MN514966) ORF encoding an 8.9-kDa protein. Alignments show that deletion in this region resulted in a premature stop codon and a truncated protein of 7.8 kDa in OrCoV/ISR1127 (deletion region highlighted in red). An amino acid comparison was generated using Geneious Prime software.

The full genome of oryx coronavirus sample ISR1127 (GenBank accession no. OM397541, here named OrCoV/ISR1127) is 31,013 bases long and has a 36.9% GC content, and its genome includes open reading frames (ORFs) encoding ORF1ab, NS2, NS5, and the major structural proteins of *Embecovirus*, namely, HE, S, E, M, and N (Fig. 4A; Table 2). Translation of these ORFs produces proteins that are similar in genome location and size to other embecoviruses (Table 2). As with other CoVs, we found a ribosomal frameshift sequence, 5′-UUUAAAC-3′, at positions 13325 to 13331, enabling the translation and production of polyprotein pp1a and pp1ab and its cleavage products, nonstructural proteins 1 to 16 (Table 2). In addition, we found that the OrCoV/ISR1127 genome exhibits transcription regulatory sequences (TRS), which are a conserved feature of CoVs (24) (Table 3). Our molecular analysis shows that OrCoV/ISR1127 TRS share high similarity in location and sequence to other *Embecovirus* TRS (Table 3) and in particular to those of DcCoV-HKU23 strain NV1097 (GenBank accession no. MN514966) (10). Next, we found additional ORFs in the region between the S gene and NS5 (Fig. 4B). This region in some embecoviruses, such as BCoV and bovine-like CoVs, includes ORF4a and ORF4b (25), with gene symbols BCoVgp05 and BCoVgp06, which code for 4.9- and 4.8-kDa nonstructural proteins, respectively (25). Sequence alignments of this region with DcCoV-HKU23 revealed two deletions in OrCoV/ISR1127, one of 16 bp (nt 27934 to 27949, in alignment) and one of 35 bp (nt 28011 to 28045, in alignment), respectively (Fig. 4B). These deletions resulted in two distinct ORFs encoding 7.8- and 4.7-kDa proteins (Fig. 4B). BLAST analysis of the hypothetical 4.7-kDa protein resulted in no significant matches in the database. Sequence alignment of the 7.8-kDa ORF with the corresponding 8.9-kDa ORF in DcCoV-HKU23 showed that the observed deletions in ISR1127 resulted in a stop codon and a truncated protein of 7.8 kDa in size that share a high similarity of 95.52% with DcCoV-HKU23 (Fig. 4C).

**TABLE 2:** Major ORFs and nonstructural proteins of OrCov/ISR1127 ORF1ab^*a*^

ORF/NSP	Name/main function/activity in coronaviruses	Location (nt)	Length (aa)
1ab	Replicase	201–21484	7094
nsp1	Blocks host cell translation	201–938	246
nsp2	Binds prohibitin proteins	939–2753	605
nsp3	Ubl1, Ac, ADRP, PLPro/deubiquitinase	2754–8450	1,899
nsp4	Transmembrane scaffold protein	8451–9938	496
nsp5	3CLpro	9939–10847	303
nsp6	Transmembrane scaffold protein	10848–11708	287
nsp7	Forms complex with nsp8 and binds nsp12	11709–11975	89
nsp8	Forms complex with nsp7 and binds nsp12	11976–12566	197
nsp9	RNA binding protein	12567–12896	110
nsp10	Cofactor for nsp16 and nsp14	12897–13307	137
nsp11	Small cleavage product, unknown function	13308–13349	14
nsp12	RNA-dependent RNA polymerase	13308–16090	928
nsp13	Helicase, 5′-triphosphatase	16091–17899	603
nsp14	N7 methyltransferase, exoribonuclease (ExoN)	17900–19462	521
nsp15	Endoribonuclease (NendoU)	19463–20584	374
nsp16	2′-*O*-methyltransferase	20585–21481	299
NS2	Nonstructural protein 2	21494–22330	278
HE	Hemagglutinin esterase	22342–23616	424
S	Spike glycoprotein	23631–27719	1,362
NS5	Nonstructural protein 5	28091–28420	109
E	Envelope protein	28413–28661	82
M	Membrane protein	28676–29368	230
N	Nucleocapsid protein	29378–30724	448

a ORF, open reading frame; NSP, nonstructural protein; OrCoV, *Oryx coronavirus*; Ubl1, ubiquitin-like domain; Ac acidic domain; ADRP, ADP-ribose-1′′-phosphatase; 3CLpro, 3C-like protease.

**TABLE 3:** Transcription regulatory sequences of the OrCoV/ISR1127 genome^*a*^

ORF	TRS		
	Location (nt)	Sequence	Distance to AUG (nt)
1ab	54	UCUAAAC	140
NS2	21480	UCUAAAC	7
HE	22326	ACUAAAC	9
S	23624	UCUAAAC	0
NS5	28038	GUUAAAC	46
E	28277	UCCAAAC	129
M	28666	UCCAAAC	3
N	29364	UCUAAAC	7

a TRS, transcription regulatory sequences; NS2, nonstructural protein 2; HE, hemagglutinin esterase; S, spike; E, envelope protein; M, membrane protein; N, nucleocapsid protein.

### Recombination analysis of OrCoV/ISR1127 with selected embecoviruses.

**FIG 5:**
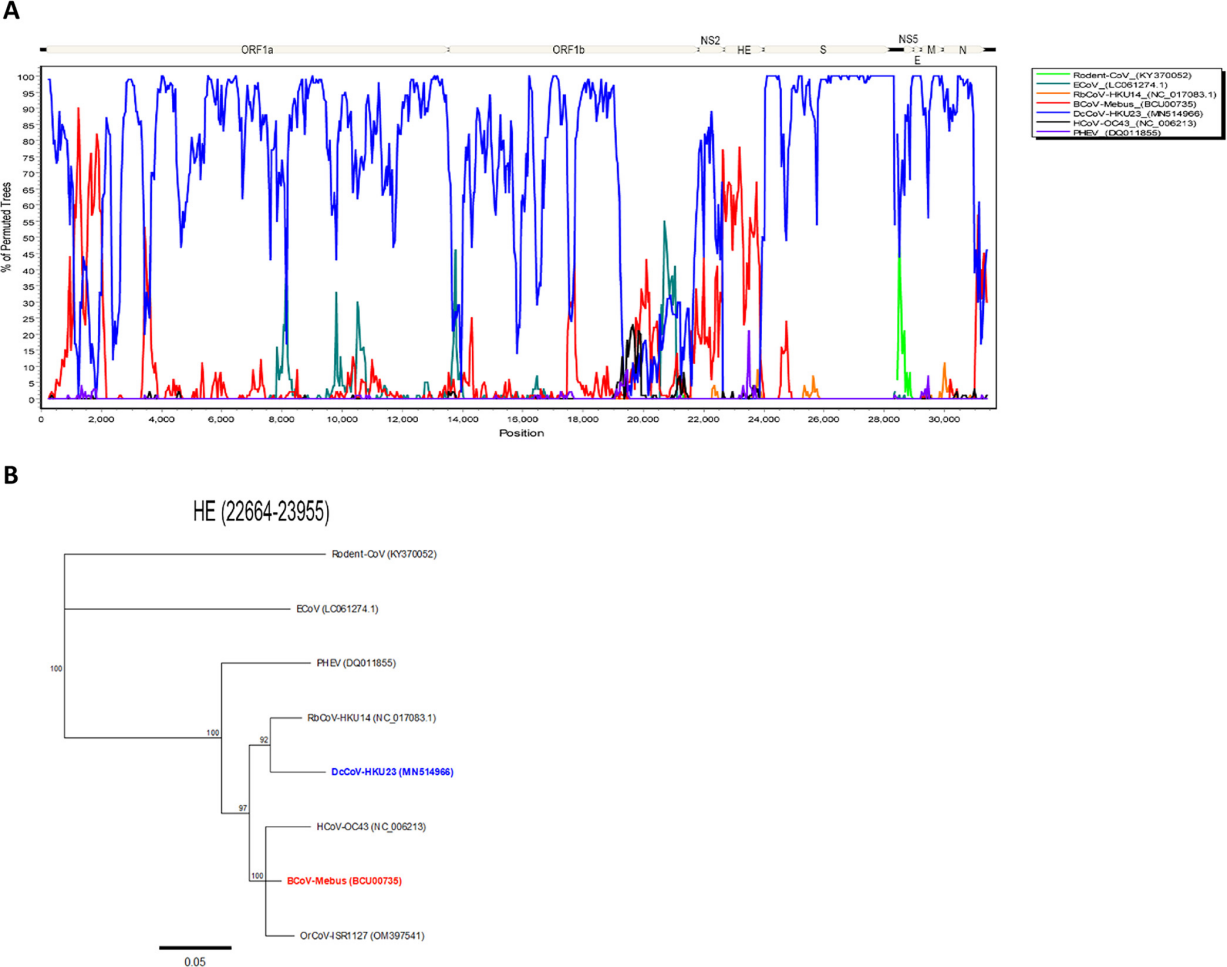
Recombination analysis of *Oryx leucoryx* coronavirus with selected embecoviruses. (A) Recombination events were explored using Bootscan analysis in Simplot software version 3.5.1. OrCoV/ISR 1127 was used as the query. (B) A phylogenetic tree of hemagglutinin esterase (HE) was generated using the neighbor-joining method with bootstrap analysis (1,000 replicates, >70%) in Geneious Prime software. Accession numbers are shown in parentheses. BCoV is highlighted in red, and DcDCoV HKU23 is highlighted in blue.

Based on our amino acid pairwise identity results, showing that the OrCoV/ISR1127 HE protein is highly similar to that in BCoV (Fig. 3C), we tested the possibility of a recombination event in OrCoV/ISR1127. To this end, we performed a Bootscan analysis of the full genome of OrCoV/ISR1127 with other selected CoVs using Simplot software (Fig. 5). While most of the positions in the OrCoV/ISR1127 genome were highly similar to those in DcCoV-HKU23, we observed a recombination signal with BCoV/mebus (GenBank accession no. BCU00735) at the HE protein (Fig. 5A). We next created a phylogenetic tree on this selected region and found that OrCoV/ISR1127 clustered with the BCoV/mebus strain and not with DcCoV-HKU23 at the HE region (Fig. 5B). Overall, these results suggest that the OrCoV/ISR1127 HE region might be the product of recombination between BCoV and DcCoV.

## DISCUSSION

In 2019, an *Oryx leucoryx* herd residing in southern Israel suffered from an outbreak of an unknown infectious agent. During this outbreak, animals experienced severe diarrhea, high morbidity rates were reported, and some animals eventually succumbed to infection (see Table S1 in the supplemental material). In an effort to identify the causative agent of this outbreak, we performed several molecular diagnostic tests based on the animals’ clinical signs. Our initial tests resulted in the identification of coronavirus in the feces of sick oryx animals (Fig. 1A and B). We next showed that this coronavirus could be isolated *in vitro* and propagated on the HRT-18G cell line, which is often used to isolate and grow animal and human CoVs *in vitro* (15, 26). We provide evidence for the presence of CoV particles in infected cells (Fig. 1E) and identify CoV RNA in tissue sections of a dead animal (Fig. 2A). Furthermore, our immunohistochemistry results show broad tissue tropism for OrCoV (Fig. 2). We found high viral loads in the intestine and abomasum of dead animals (Fig. 2B), which is correlated with severe enteric symptoms observed in animals during this outbreak. Interestingly, we found CoV antigen in the heart of a dead animal and particularly located in the Purkinje fibers (Fig. 2D). The presence of CoVs in the heart is well documented for SARS-CoV-2 infection in humans, and cardiac arrhythmias have been observed during SARS-CoV-2 infection (27, 28). Still, a direct link between CoVs and an irregular heart rhythm has yet to be established. Our findings of CoV antigen in Purkinje fibers in the heart of a dead oryx might provide a potential link to explain arrhythmia during CoV infection. Although we cannot rule out the possible contribution of other pathogens to the severe enteric disease and mortality observed in the oryx herd, our finding of coronavirus in these animals’ feces and tissues (Fig. 1 and 2) is highly characteristic of enteric infections caused by CoVs (29).

Coronavirus spike protein (S) is essential for the attachment and entry of the virus to target cells and is crucial in virus pathogenicity (30). The S protein is often used to determine the genetic variability, evolution, and emergence of novel strains and variants in both human and animal hosts (15, 23, 31, 32). Based on our initial qPCR test, detecting the BCoV nucleocapsid gene, we PCR amplified the S1 region of coronavirus isolated from oryx fecal sample ISR1127 (Table S2). Our S1 phylogenetic analysis suggested that ISR1127 is genetically different from our local BCoV strains (Fig. 3A), and BLAST analysis showed that OrCoV/ISR1127 is most similar to DcCoV-HKU23. We then sequenced its full genome and performed a phylogenetic analysis and amino acid comparisons with other CoV strains (Fig. 3B and C). Full-genome sequencing revealed that OrCoV/ISR1127 is most similar to DcCoV-HKU23 and shares more identity with dromedary coronavirus than with bovine coronavirus or bovine-like coronavirus, often isolated from wild animals (Fig. 3) (9). OrCov/ISR1127 shares an overall genome identity of 97.93% with DcCoV-HKU23, a member of *Embecovirus*, and based on the amino acid identity of the replicase conserved domain, OrCoV/ISR1127 belongs to the subgenus *Embecovirus* and the *Betacoronavirus 1* species (Table 1). Additionally, OrCoV/ISR1127 shares high amino acid identity with *Embecovirus* structural proteins, NSPs, and TRS and locations and overall shares the same genome organization (Tables 2 and 3; Fig. 4).

Furthermore, we found distinct ORFs in the region between the S gene and NS5 encoding 7.8- and 4.7-kDa proteins, respectively (Fig. 4B and C). This region contains ORF4a and ORF4b in BCoV, and it is divergent between CoVs (33). A recent study reported genetic diversity in this region among DcCoV-HKU23 isolates and suggested that recombination events might explain the differences in ORF size and protein products found in this region (33). Interestingly, we discovered that OrCoV/ISR1127 ORF4a, which encodes the 7.8-kDa protein, is similar to the DcCoV-HKU23/NV1097 protein of 8.9 kDa, but due to a deletion event that resulted in a stop codon, it is smaller (Fig. 4C). Although studies showed that these proteins are not essential for CoV replication, they might have a role in viral tropism (34).

Recombination events between CoV species have been previously described (33, 35). To assess the possibility of recombination events in OrCoV/ISR1127, we screened the OrCoV/ISR1127 full genome and compared it to the genomes of selected embecoviruses (Fig. 5). We provide evidence for a potential recombination event in the HE gene between BCoV and DcCoV in oryx (Fig. 5A and B). This result might suggest the existence of coinfection of BCoV and DcCoV in the oryx host, which might be possible due to the fact that both animals inhabit this geographic region and the viruses potentially could circulate in the bovine and camel populations. Spillover events of different CoVs are a known phenomenon (36, 37). Moreover, the observation of spillover events of BCoV and DcCoV to infect other hosts have been recently reported (38, 39). The recent outbreak in the oryx herd and our molecular findings could support a recent spillover event of coronavirus into the oryx herd residing in southern Israel. However, the ability of this virus to spread in the oryx herd might suggest an adaptation to this host and an old introduction of this viral agent.

Based on our results, we conclude that the above-isolated coronavirus is a newly identified *Betacoronavirus*, in the subgenus *Embecovirus*, and we therefore suggest naming it oryx coronavirus (OrCoV).

## MATERIALS AND METHODS

### Clinical samples.

From January to March 2019, *Oryx leucoryx* animals in a herd residing in southern Israel suffered from severe diarrhea. In an attempt to identify the causative agents of this illness, rectal swabs from five sick animals, named ISR1127 samples 1 to 5, and tissues from a dead animal named ISR1117 were sent to the Kimron Veterinary Institute for diagnosis. Additional samples for monitoring were collected for diagnosis until January 2021 (see Table S1 in the supplemental material). Samples were further processed as follows. Rectal swabs were suspended in 2 mL of PBS, incubated for 20 min at room temperature, and then centrifuged at 1,000 rpm for 10 min. Animal organs/tissue samples were homogenized in PBS (1 g of tissue in 9 mL of PBS) and then centrifuged at 4,500 rpm for 10 min. Supernatant from each sample was kept at −80°C for future analysis. All samples used in this study were samples that arrived for routine diagnosis and were approved for further analysis under Kimron Veterinary Institute guidelines and ethics.

### RNA extraction and molecular detection of coronavirus in oryx samples.

Processed samples were initially tested for the presence of coronavirus using a pancoronavirus nested PCR targeting the RdRp gene as previously published (Table S2) (16). Briefly, 100 μL of each sample was used to extract RNA using the MagMAX core nucleic acid purification kit (Applied Biosystems, Carlsbad, CA, USA) and KingFisher duo prime (Thermo Scientific, Carlsbad, CA, USA), according to the manufacturers’ instructions. Purified RNA was used to perform the first round of RT-PCR using the Quanta qScript XLT one-step RT-PCR kit (Quantabio, Beverly, MA, USA). The first-round product was used for a second PCR using Dream Taq green master mix (Thermo Scientific, Carlsbad, CA, USA). The final product of 440 bp was gel purified using a MEGAquick-spin plus kit (iNtRON, Seoul, South Korea) and analyzed by Sanger sequencing. In addition, purified RNA was tested for the presence of BCoV RNA by targeting the BCoV nucleocapsid gene as previously described (Table S2) (15, 40). Briefly, purified RNA was used to perform RT-qPCR using the Quanta qScript XLT one-step RT-qPCR ToughMix kit (Quantabio, Beverly, MA, USA) and analyzed using the Bio-Rad CFX 96 real-time detection system (Bio-Rad, Hercules, CA, USA).

### Virus isolation and propagation in cell culture.

Following molecular detection of coronavirus, positive fecal samples were filtered using a 0.45-μm membrane and used for virus isolation by serial passaging on the human rectal tumor cell line HRT-18G (ATCC CRL11663). To this end, HRT-18G cells were grown in 25-cm^2^ flasks (Corning, NY, USA) in Dulbecco’s modified Eagle’s medium (DMEM) (ATCC 30-2002) supplemented with 10% fetal bovine serum (Gibco, Invitrogen, CA, USA), 0.1 mg/mL streptomycin, and 100 U/mL penicillin (Biological Industries, Israel). Cells were kept at 37°C with 5% CO_2_ to reach a confluent monolayer. Next, 1 mL from the postfiltered sample was applied to the HRT-18G monolayer and incubated for 1 h at 37°C and 5% CO_2_. Following adsorption, cells were washed with PBS and resuspended in DMEM with no serum. Cells were inspected daily for the presence of a CPE. For replication assays, we infected HRT-18G cells with ISR1127 sample 1 (here called ISR1127 for simplicity) and collected cells at different time points, extracted RNA, and performed RT-qPCR as described above. All experiments with isolated viruses and human cells were carried out under restricted biosafety level 2 measurements.

### TEM.

HRT-18G cells were infected with ISR1127 for 48 h. Then, the infected cells were collected and fixed with 2% formaldehyde and 2.5% glutaraldehyde in 0.1 M cacodylate buffer, pH 7.4, at room temperature for 2 h, washed, and postfixed with 1% osmium tetroxide in the same buffer for 1 h at room temperature; the samples were then dehydrated in a graded ethanol series and embedded in Epon. Thin sections (70 to 90 nm) were prepared using an Ultracut UCT microtome (Leica), followed by poststaining with 2% uranylacetate and Reynold’s lead citrate, and viewed in a JEOL JEM-1400 Plus transmission electron microscope (TEM) (JEOL, Tokyo, Japan) operating at 100 kV, equipped with an Orius SC600 charge-coupled-device camera (Gatan, Abingdon, United Kingdom) and using Gatan Microscopy Suite program (DigitalMicrograph, Gatan, UK).

### Extraction and detection of *Oryx leucoryx* coronavirus RNA from FFPE tissues sections.

Tissue sections (10 μm) were prepared from FFPE blocks prepared from *Oryx leucoryx* ISR1117 sample tissues. Tissue sections were placed into 1.5-mL tubes and then resuspended with deparaffinization solution (Qiagen, Hilden, Germany) according to the manufacturer’s instructions. Tissue RNA was extracted using an Rneasy FFPE kit (Qiagen, Hilden, Germany) and used to perform RT-qPCR for BCoV detection as described above.

### IHC.

IHC analysis was performed on FFPE *Oryx leucoryx* blocks from one dead animal, ISR1117, which died during the outbreak in 2019. IHC staining was performed on 4-μm sections by using the Leica Bond-Max system (Leica Biosystems Newcastle Ltd., UK). Sections were dewaxed and pretreated with epitope retrieval solution (ER1, Leica Biosystems Newcastle Ltd., UK), followed by 30 min of incubation with 1:100 monoclonal anti-nucleocapsid antibody of BCoV (BC 6-4-A; RTI, SD, USA) (Fig. S1A to C) or with 1:100 mouse IgG1 isotype control (BP0083; InVivoPlus) by using the same protocol (Fig. S1D). The Leica Refine HRP kit (Leica Biosystems Newcastle Ltd., UK) was used for detection and counterstaining with hematoxylin. Antibody calibration and specificity were determined by negative controls as described in the legend to Fig. S1A to D. Slides were analyzed using Nikon microscopy and NES elements software (Nikon Eclipse Ci).

### PCR and spike S1 sequencing.

Total RNA was extracted from the ISR1127 source sample as described above and used to amplify the S1 region of the spike glycoprotein, using the Quanta qScript XLT one-step RT-PCR kit (Quantabio, Beverly, MA, USA). For S1 amplification, we used a six-primer set previously described, with slight modification to allow the amplification of nucleotides 1 to 2731 of the S glycoprotein of ISR1127 (Table S2) (23). RT-PCR products were loaded on a 1.5% agarose gel, and corresponding bands were gel extracted and purified using a MEGAquick-spin plus kit (iNtRON, Seoul, South Korea). Purified PCR products were verified by Sanger sequencing (Hylabs, Rehovot, Israel). The sequenced S1 region of ISR1127 was further used in BLAST and phylogenetic analysis.

### Whole genome sequencing.

Total RNA was extracted from HRT-18G cells infected with isolate ISR1127 for 48 h as described above. RNA sequencing was performed at Genotypic Technology Pvt. Ltd. (Bangalore, India) according to company protocols. Briefly, RNA sequencing libraries were prepared with an Illumina-compatible NEBNext Ultra II directional RNA library prep kit (New England BioLabs, MA, USA). Fragmented and primed RNA was subjected to first-strand synthesis, followed by second-strand synthesis. The double-stranded cDNA was purified using JetSeq beads (Bioline; catalog no. BIO-68031). Purified cDNA was end repaired, adenylated, and ligated to Illumina multiplex barcode adapters as described in the NEBNext Ultra II directional RNA library prep protocol, followed by second-strand excision using USER enzyme at 37°C for 15 min. Illumina adapter-ligated cDNA was purified using JetSeq beads and subjected to 10 to 12 cycles for indexing (98°C for 30 s, cycling [98°C for 10 s, 65°C for 75 s], and 65°C for 5 min) to enrich the adapter-ligated fragments. The final PCR product (sequencing libraries) was purified with JetSeq beads, followed by a library quality control check. Illumina-compatible sequencing libraries were quantified using a Qubit fluorometer (Thermo Fisher Scientific, MA, USA), and the fragment size distribution was analyzed on an Agilent 2200 TapeStation. The libraries showed optimal quantity in nanograms and quality with respect to size distribution, were pooled in equimolar quantities, and were sequenced on an Illumina HiSeq XTen sequencer (Illumina, San Diego, USA) using 150-bp paired-end chemistry and HiSeq XTen SBS reagents. Raw reads were mapped to DcCoV-HKU23 (GenBank accession no. MN514966), and a coverage plot was generated to verify coverage along the full genome (Fig. S2A). Areas of interest were further verified by target amplification and Sanger sequencing using additional isolates (Fig. S2B, isolates ISR1127 samples 2, 3, and 5). The ISR1127 full genome (GenBank accession no. OM397541) was generated based on a consensus sequence using Geneious Prime software tools (Biomatters, CA, USA).

### Molecular and phylogenetic analysis.

The *Oryx leucoryx* coronavirus full genome and selected *Betacoronaviruses* strains were aligned with the Geneious alignment tool using Geneious Prime software (Biomatters, CA, USA). These alignments were used to create a phylogenetic tree. A phylogenetic tree was produced based on the neighbor-joining method and bootstrapping with 1,000 replicates and a support threshold of 70%. The deduced amino acid sequences for the different ORFs were then aligned to the DcCoV (GenBank accession no. MN514966) strain as a reference strain, and amino acid differences were identified by the protein alignment tool in Geneious Prime software.

Recombination analysis was performed using Simplot software version 3.5.1 (41). We used an alignment of selected embecoviruses with oryx coronavirus sample ISR1127 to perform a Bootscan analysis using Simplot with a sliding window of 500 bp and a 50-bp step size. Oryx coronavirus sample ISR1127 served as a query.

### Data availability.

The complete genome of *Oryx leucoryx* coronavirus isolate ISR1127, sequenced in this study, is openly available at the National Center for Biotechnology Information (NCBI) database under accession no. OM397541.
