# Supplementary figures and images for: Identification, Isolation, and Molecular Characterization of Betacoronavirus in Oryx leucoryx
Source: Microbiol Spectr. 2023 Jul 10;11(4):e04848-22. doi: 10.1128/spectrum.04848-22 (PMC10433975; doi:10.1128/spectrum.04848-22)

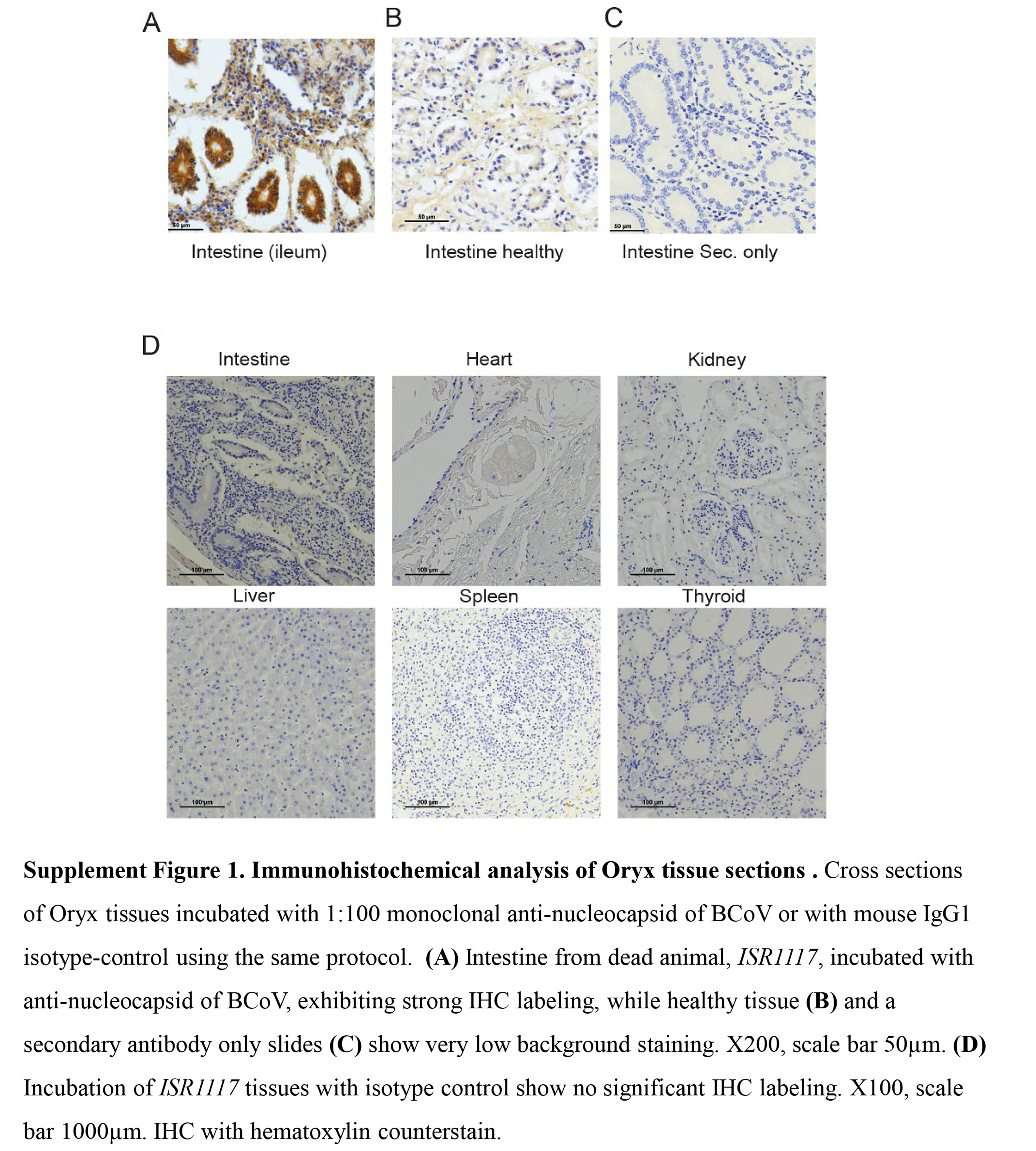

Supplement: Supplemental file 1 — Supplemental material. Download spectrum.04848-22-s0001.tif, TIF file, 10.9 MB [file spectrum.04848-22-s0001.tif]

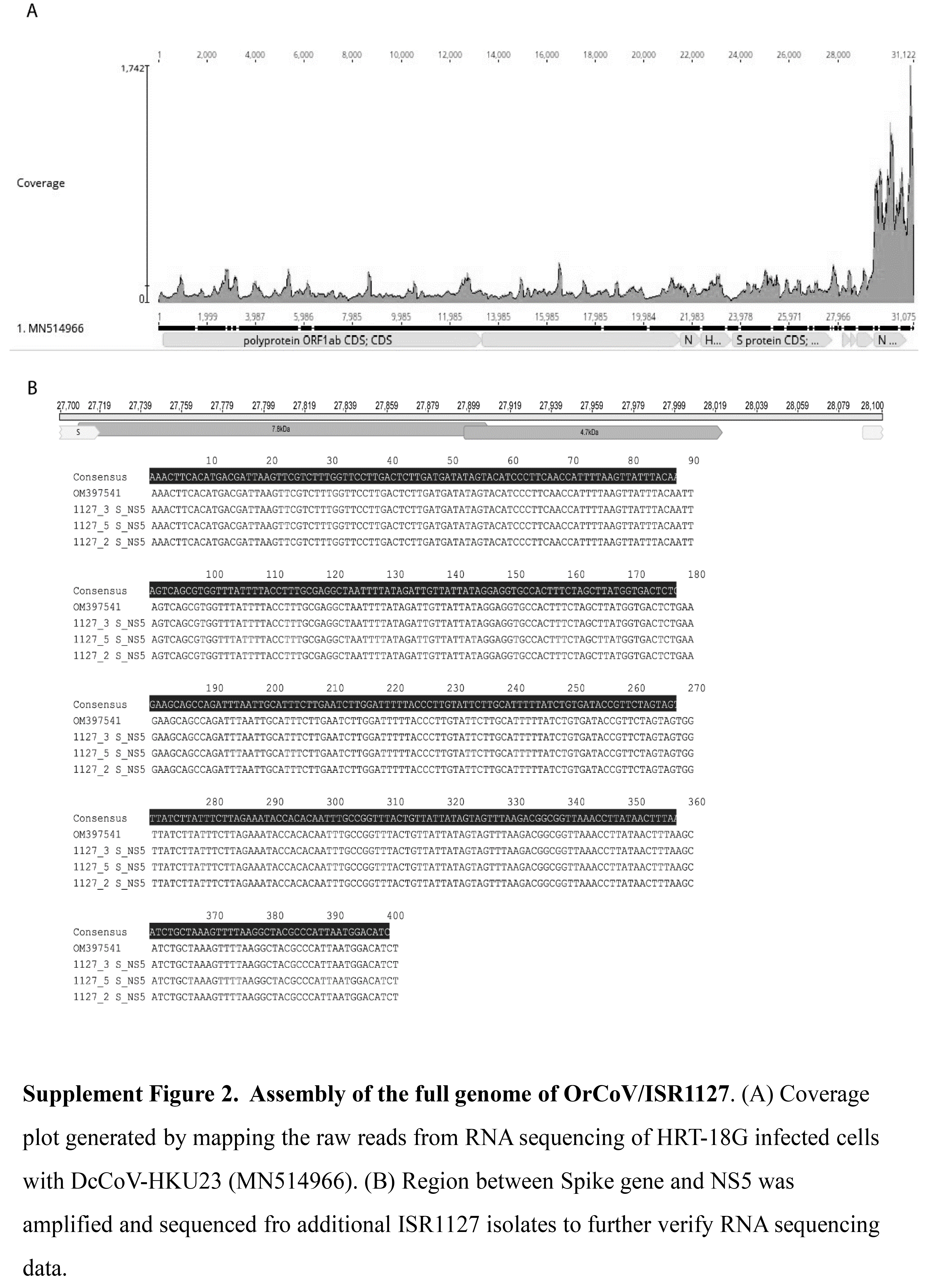

Supplement: Supplemental file 2 — Supplemental material. Download spectrum.04848-22-s0002.tif, TIF file, 3.8 MB [file spectrum.04848-22-s0002.tif]

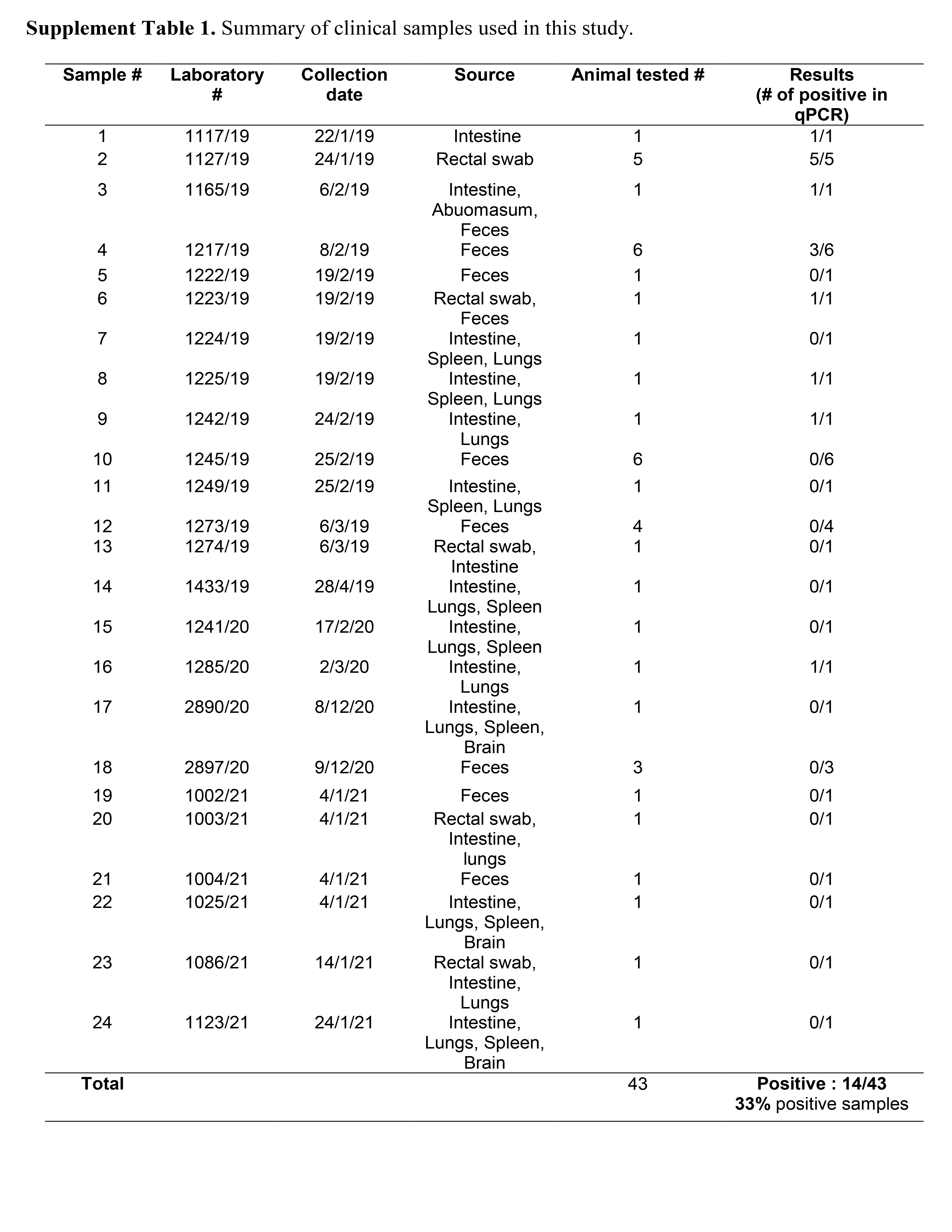

Supplement: Supplemental file 3 — Supplemental material. Download spectrum.04848-22-s0003.tif, TIF file, 0.7 MB [file spectrum.04848-22-s0003.tif]

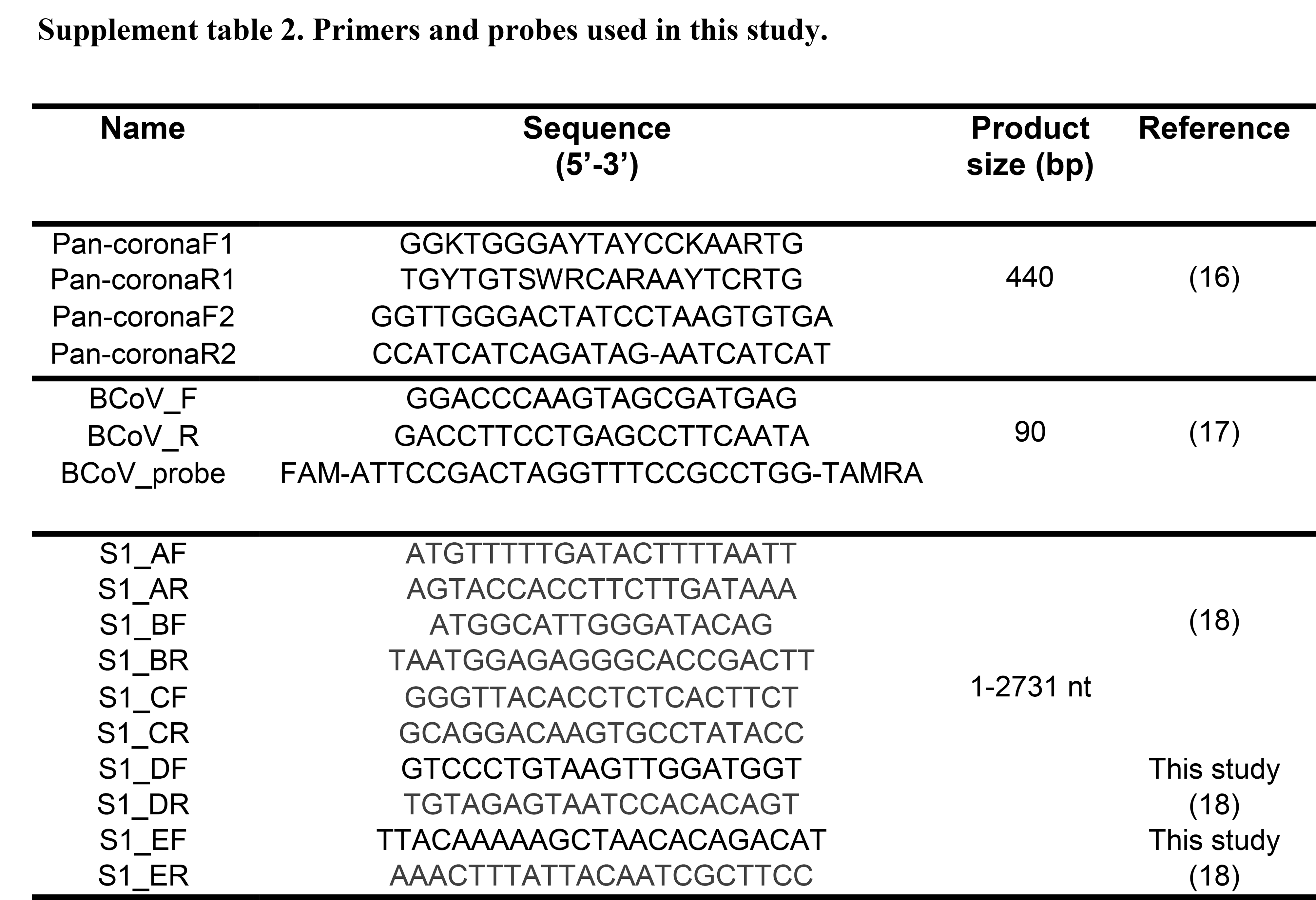

Supplement: Supplemental file 4 — Supplemental material. Download spectrum.04848-22-s0004.tif, TIF file, 0.9 MB [file spectrum.04848-22-s0004.tif]
